# Reframing the Overdose Crisis: Stigma, Industry Influence, and the Politics of Abuse-Deterrent Opioids

**DOI:** 10.1177/27551938251378941

**Published:** 2025-09-26

**Authors:** Daniel Eisenkraft Klein, Quinn Grundy, Benjamin Hawkins, Robert Schwartz

**Affiliations:** 1 Dalla Lana School of Public Health, 47965 University of Toronto, Toronto, Canada; 2 Program On Regulation, Therapeutics And Law (PORTAL), Division of Pharmacoepidemiology and Pharmacoeconomics, Brigham and Women's Hospital, Harvard Medical School, Boston, MA, USA; 3 Lawrence Bloomberg Faculty of Nursing, 47965 University of Toronto, Toronto, Canada; 4 MRC Epidemiology Unit, 7938 University of Cambridge, Cambridge, UK

**Keywords:** abuse-deterrent opioids, overdose crisis, opioid crisis, stigma, policy framing, opioid industry, opioids, oxycontin

## Abstract

Between 2013 and 2017, Canadian federal policymakers grappled with mandating abuse-deterrent formulations (ADFs) for oxycodone products as a response to the overdose crisis. Marketed as a safeguard against misuse and diversion, ADFs promised a technological fix to opioid-related harms, yet their population-level effectiveness remained contested. This study systematically analyzes federal parliamentary debates and committee hearings, identifying key arguments in framings to support or oppose ADF mandates. Proponents framed the crisis through the lens of individual misuse, positioning ADFs as pharmaceutical safeguards that protected “legitimate” patients while curbing illicit opioid use. Opponents challenged ADFs’ effectiveness, highlighted Purdue Pharma's role in the crisis, and warned of unintended consequences, including shifts to more dangerous illicit markets. These discursive struggles reinforced a bifurcation between “legitimate” and “illegitimate” opioid use, shaping perceptions of responsibility, medical necessity, and the scope of appropriate intervention. Divergent framings reflected deeper ideological fissures over the etiology of the overdose crisis and who should be considered a justifiable opioid patient. By demonstrating how ADF debates entrenched a dichotomy between acceptable and unacceptable opioid use, this study advances theories of problem framing to demonstrate how policy debates actively shape regulatory paradigms and the boundaries of acceptable government intervention.

In 2012, with the company's patents set to expire, Purdue Canada removed its standard controlled-release oxycodone product, OxyContin, from the Canadian market and replaced it with its reformulated OxyNEO, an abuse-deterrent formulation (ADF) of OxyContin. Notably, Purdue introduced OxyNEO in Canada two years after launching its ADF in the United States, a delay that suggests Purdue may have prioritized revenue over potential public health benefits.^1^ Like most ADFs, OxyNEO incorporated mechanical barriers designed to make crushing or dissolving the pill more difficult, thereby making it harder to achieve a “rapid high”.^2^

Following the expiry of Purdue's patent on OxyContin (Patent ‘738) in 2012, six Canadian drug manufacturers began producing generic versions of controlled-release oxycodone. Despite Canadian prescribers writing more than 15 million opioid prescriptions annually (more than one for every two Canadians on average) by 2013, Purdue's market share began to decline once their patent had expired.^3^ Without a mandate requiring opioids to be sold as ADFs, generic forms of non-ADF controlled-release oxycodone remained on the market.

Between 2013 and 2017, Canadian policymakers debated mandating ADFs. Health Canada proposed regulations in 2015, but the Liberal government abandoned them in 2016. A Private Member's Bill (C-307) in 2016–2017 also failed, and the issue faded from the policy agenda. Throughout, ADFs were endorsed as a valuable component of a broader overall strategy against the overdose crisis by both Canadian health policy researchers and government agencies,^4,5^ with mandates extensively debated and discussed in Canadian media.^6,7^ While some Canadian policy researchers endorsed ADF mandates, these endorsements did not necessarily represent a broad research consensus, as certain prominent supportive voices, such as Skinner and colleagues^5^ were affiliated with market-oriented organizations closely linked to pharmaceutical industry interests.

There have been numerous studies evaluating the effectiveness of ADFs as a policy intervention.^2,8,9^ Yet despite extensive separate bodies of literature on the clinical efficacy of ADFs, little attention has been paid to how positions about ADFs served to reveal perceptions of the causes and solutions of the crisis. That ADFs served as among the most prominent policy proposals to the overdose crisis in both Canada and the United States^2,10^ represents a critical gap.

In this vein, this study critically examines the framings of ADFs within discussions in Canadian Parliamentary Committees and the Canadian House of Commons. Focusing on presentations of ADFs in formal policy settings provides important insights into how policy solutions simultaneously serve to characterize the overdose crisis, the opioid industry, and the roots of substance abuse more generally. These findings contribute to understandings of how public health policy makers resolve problems while simultaneously shaping how they are defined.

## Background and Research Context

### The Structure of Canadian Health Policy Making

Understanding the Canadian institutional landscape is crucial for analyzing how ADFs were framed in political discourse and how different actors shaped the debate around opioid regulation in Canada. The Canadian federal legislative system consists of two chambers: the elected House of Commons, featuring a wide ideological spectrum due to party-based elections, and the appointed Senate, whose members are selected by the sitting Prime Minister, typically resulting in a narrower range of political perspectives.^11^ At the time of these debates, the Liberal Party, led by Prime Minister Justin Trudeau, held a majority government, and therefore had significant influence over legislative outcomes. The Conservative Party of Canada (CPC; Canada's primary center-right party) formed the Official Opposition, with smaller parties such as the New Democratic Party (NDP; Canada's primary left-leaning, social democratic party) and the Bloc Québécois (BQ; a Quebec nationalist party) also participating in debates. As the federal agency responsible for approving and monitoring pharmaceuticals, Health Canada plays a central role in public health regulation, including the evaluation of drug safety, efficacy, and labeling.^12^ Given Health Canada's role as the federal agency overseeing pharmaceutical approvals and safety standards, ADF proposals were subject to review not only by government regulators but also by Canadian Parliamentary Committees, which assessed these proposals through expert testimony, lobbying efforts, and political debate before advancing them for legislation.^13^

### OxyContin and Abuse-Deterrent Formulations

Standard (non-ADF) OxyContin is a controlled-release formulation of oxycodone designed to provide extended pain relief. Although Purdue marketed OxyContin as providing 12-h pain relief, its real-world effectiveness was closer to 8 h.^14^ However, this controlled-release mechanism can be bypassed through tampering, leading to a rapid release of the drug.^15^ Common tampering methods of oxycodone include chewing, snorting, or intravenous injection, all of which generally facilitate faster absorption of the drug, thereby allowing the medication to be released all at once and enhancing the experience of euphoria and risk of overdose from the product.^15^

To counteract this form of tampering, ADFs are specifically designed to withstand physical and chemical manipulation, preventing the rapid release and enhanced euphoric effects associated with misuse. ADFs can encompass a variety of mechanisms, including barrier agents, antagonist combinations, aversive agents, and prodrug elements.^15^ Physical and chemical barriers—the most common form of controlled-release oxycodone ADFs (henceforth referred to as “OxyNEO,” the brand name)—are designed to withstand cutting, grinding, or pulverizing of the drugs. When crushed, OxyNeo would transform into a “flat pancake” instead of a powder. When wet, it turned into a gel, making it challenging to inhale or inject.

### Purdue Canada's Promotion of OxyNeo in Canada

By the time Purdue Canada (Purdue) introduced OxyNeo in February 2012, the company was already facing significant scrutiny as a central architect of the opioid crisis.^16^ Despite this scrutiny, Purdue made a concerted effort in Canada to promote ADFs as an essential solution to the overdose crisis. This multifaceted strategy encompassed a spectrum of influence-building tactics, including advertising in medical journals, attendance at medical conferences, journal funding and writing, policymaker lobbying, and marketing. It promoted OxyNEO through advertising and labelled the product as “far safer” in brochures; however, this label came with an asterisk and, in small print at the bottom, a disclaimer noted that “the clinical significance of these results has not yet been established.” Purdue also ran eight half- and full-page ads in the *Canadian Medical Association Journal* in 2014, promoting OxyNEO as “a recommended first-line option for severe pain”.^7^ At the Canadian Pain Society conference in 2015, which hosted physicians from across the country, Purdue had a prominent booth flanked by large advertisements for OxyNEO, with employees handing out promotional brochures to physicians and other attendees.^7^

### Evidence for and Against Mandating OxyContin Abuse-Deterrent Formulations

Mandating ADFs was a heavily contested policy solution to the overdose crisis in large part because of the mixed and conflicting evidence for the effectiveness of ADFs in mitigating the harms of opioid use. On one hand, evidence indicated that ADFs were effective in preventing particular forms of tampering, including injection and snorting.^17^ As a result, though injection, snorting, and smoking were not the primary main form of extra-medical use, these forms of tampering would likely be discouraged if controlled-release oxycodone was only available in an ADF-form and individuals used the ADF product as intended. As such, the reformulations of OxyContin were associated with significant reductions in certain forms of harms related to controlled-release oxycodone use, including reports to poison control, overdose deaths, and past-month use.^2,18^ Other evidence suggested that certain measures of non-oral use that required tampering, including injection, snorting, and smoking, also declined with the reformulation.^18^

On the other hand, despite ADFs’ ability to deter some forms of tampering, the capacity for mandating ADFs to make a significant positive impact on overall population harms was heavily disputed; non-oral use of oral formulations was not a factor in the majority of Canadian opioid-related deaths, instances of nonmedical opioid use, or admissions to treatment centres.^19,20^ In addition, many of those who were previously tampering with OxyContin were able to bypass the new formulation (e.g., by microwaving the pill), while others merely transitioned to ingesting it intact, but continued using it without medical approval.^2^ The mandating of ADFs in the United States in 2010 appears to have pushed many opioid users to seek opioids within illicit markets and unregulated opioids.^21,22^ ADFs were also far more expensive than standard opioids; between 2010 and 2012, mandating ADFs led to between a five and 15-fold increase in cost in Medicare spending in the United States.^23^

Taken together, these conflicting findings underscore how ADFs had the potential to be positioned to be seen as either a targeted harm reduction measure aimed at preventing opioid misuse or as an ineffective and potentially harmful intervention that failed to address the structural drivers of the overdose crisis. These competing perspectives underscore the importance of examining how ADFs were framed within formal policy discussions. The following section outlines the methods used to conduct this analysis.

### Theoretical Framework: Policy Framing Theory

This study's approach is rooted in policy framing theory. Framing theory assumes that policy problems and their proposed solutions are inherently contestable; historical, social, and cultural contexts are vital to understanding policies; and the performative components of policy formation—how policies are presented and discussed—are of significant importance to policy analysis.^24,25^ Moreover, policy is not made by “rational” actors and decision making does not occur through a set of discreet and linear stages, but rather a highly political process wherein a range of factors determine the outcome of a policy.^26^ In framing theory, every policy is shaped by a particular understanding of the issue, which opens up the possibility for certain solutions while excluding others.^25^ Policy framings thus not only reflect underlying assumptions about the nature of a problem but also actively construct their meanings and influence the range of policy responses deemed viable. Framing plays a pivotal role in determining whether an issue gains attention,^27^ is identified as a policy problem requiring government attention, and how that problem is addressed.^28^ Moreover, it shapes public perceptions and attitudes toward policy decisions.^29^

There are various definitions of frames and framings, reflecting the complexity and interdisciplinary nature of framing theory. This study draws on Rein and Schön's^30^ *frame-critical policy analysis*. We specifically employ Rein and Schön's framing theory due to its utility in critically examining how policy actors explicitly connect problem definitions to policy actions, thus enabling deeper analysis of the dynamic debates surrounding ADF mandates. In particular, Rein and Schön situate meaning within their historical and sociopolitical contexts, which allows for examination of why and how particular groups’ interpretations are considered over others and how they serve different policy stakeholders’ interests. This approach also allows for an account of the broader forces that shape actors’ perspectives and, consequently, policy beliefs.

Rein and Schön define a *policy frame* as a normative and prescriptive narrative of a policy problem that thereby also provides an appropriate course of action to address this problem. The authors argued that through the social construction of the problem, framing “provides conceptual coherence, a direction for action, a basis for persuasion, and a framework for the collection and analysis of data—order, action, rhetoric, and analysis”.^30^(p. 153). This language continued Entman's^31^ and others’ conception of framing as simultaneously defining an issue and suggesting a solution.

Framings are of particular importance to the overdose crisis, as Laugesen and Patashnik^32^ argue: “There is no one correct or natural way to understand the opioid epidemic. How people understand it—and how they apprise the benefits and costs of different ways to address it—are shaped by the political context in which discussions about the epidemic take place. It is clear that in such discussions words matter, and that it is impossible to even conceive of the politics or policy response without considering the narrative and framing, or how we describe it (pain management, drug abuse, public health epidemic)” (p. 366). Framing theory served as an essential framework to explore the dynamic and contested nature of how ADFs were positioned within the broader discourse of the opioid overdose crisis. Given that framing not only delineates policy problems and solutions but also actively influences the epistemic boundaries of public health discourse, this study explores how ADFs were positioned within policy discussions and what these framings reveal about underlying assumptions regarding opioid use, industry responsibility, and patient legitimacy. Specifically, it seeks to understand how policymakers constructed ADFs as a policy solution, how these constructions reflected competing narratives regarding the etiology of the overdose crisis, and how they shaped the perceived boundaries of permissible policy interventions. To investigate these questions, the study draws on a systematic analysis of parliamentary committee discussions and House of Commons debates, as outlined in the subsequent section on data collection and analysis.

## Methods and Data

### Data Sources

All data was collected via OurCommons.ca, a Government of Canada website that provides a searchable database of Hansard (Debates of the House of Commons), House and Senate Committee Evidence, and Board of Internal Economy (BOIE) transcripts. All data is openly accessible via these public Canadian databases. House of Commons debates were found via the Hansard Index (Hansard), a verbatim transcription of what is said by Members of Parliament in the House of Commons.^33^ The Hansard provides an avenue to understand public and policymaker discourse in a systematic way and is useful in understanding how policymakers and the public view effective and acceptable responses to health problems.^34,35^

### Data Collection

We pursued data collection as an iterative process, consistent with the principles of interpretive study design.^36^ We began by searching the entire database with terms related to ADFs: “abuse-deterrent(s),” “abuse-deterrence,” “tamper-resistant,” “tamper-resistance,” “tamper,” and “oxyneo.” Only English-language results were included, as French speeches were translated before entry. No date restrictions were applied. Upon completion, this strategy identified three primary parliamentary settings in which policymakers discussed and heard evidence on mandating opioid ADFs:

- House of Commons’ Standing Committee on Health's “Hearings on Government's Role in Addressing Prescription Drug Abuse” (October 30, 2013–April 1, 2014)
- The Standing Senate Social Affairs, Science and Technology Committee's “Special Study on Prescription Pharmaceuticals in Canada” (January 29, 2014–April 30, 2014)
- Canadian House of Commons Canada Debates on Bill C-307: An Act to Amend the Controlled Drugs and Substances Act (tamper resistance and abuse deterrence; September 28, 2016–April 5, 2017)

The House of Commons Standing Committee on Health (HESA) held 10 meetings and heard from witnesses with varying views: nine supported ADFs, five were skeptical, and four expressed mixed opinions. The Committee's final report recommended that Health Canada consider the merits of ADFs but stopped short of suggesting a mandate. Opposition parties went further, with the New Democratic Party calling for regulations requiring ADFs for all addictive drugs, while the Liberals recommended banning generic OxyContin and replacing it with tamper-resistant OxyNEO for specific federal programs. The Senate Committee, which met 17 times between January and April 2014, heard similarly diverse testimony. Unlike the House, the Senate's final report recommended that all prescription opioids with abuse potential incorporate ADFs as a condition of market approval, with no dissent noted. The results from these two committees’ reports and hearings demonstrated highly similar framing strategies, particularly in contrast to the House of Commons debates, and are therefore organized and presented in this study together in the first section.

**Figure 1.**
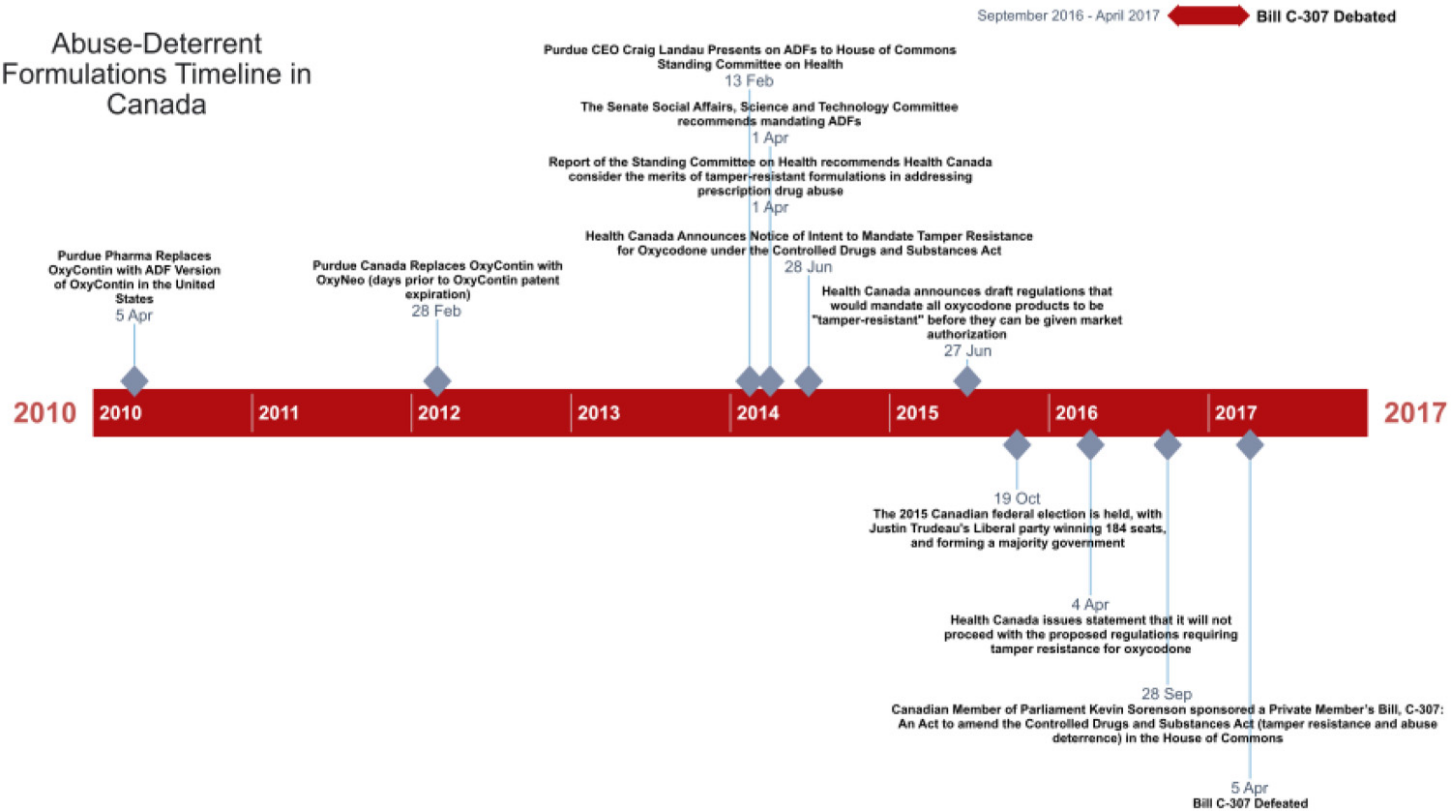
Abuse-Deterrent Formulations (ADF) Federal Discussions Timeline in Canada.

Finally, we analyzed the House of Commons Debates on Bill C-307: An Act to Amend the Controlled Drugs and Substances Act (tamper resistance and abuse deterrence) between September 28, 2016, and April 5, 2017. These debates showcased a range of arguments from multiple Members of Parliament (MPs), both advocating for and opposing the proposed mandate. The timeline of these three policy arenas can be seen in Figure 1.

### Data Analysis

The first author independently conducted all coding and qualitative analysis. We employed framing analysis to explore how stakeholders constructed problem definitions and policy solutions to shape their narratives about prescription opioids and the overdose crisis. Our approach drew on Rein and Schön's^30^ concept of frames as “a perspective from which an amorphous, ill-defined, problematic situation can be made sense of and acted on” (p. 146). By applying this method, we examined how stakeholders strategically framed issues to influence public perceptions and policy debates. While coding for framings, particular attention was paid to how stakeholders framed both ADFs and the crisis itself (e.g., “prescribing problems” or “opportunity to restrict opioid marketing”). During coding, we also made note of whether actors were supportive, opposed, or presented mixed opinions in relation to ADFs. All attributions of responsibility for and solutions to the crisis were noted. Once completed, a set of core framings was developed, as outlined in the next section.

## Findings

This section presents the primary framings of ADFs by both supportive and opposing stakeholders during committee proceedings, as summarized in Tables 1 and 2. Overall, proponents of ADF mandates demonstrated a marked evolution in their framing, shifting from a wide range of rationales for support toward a highly coordinated narrative emphasizing “misuse, abuse, and diversion” and positioning ADFs as innovative and essential tools in addressing the opioid crisis. Opponents maintained consistent skepticism, combining technical critiques of ADF efficacy with distrust of Purdue's history.

**Table 1.** Abuse-Deterrent Formulations (ADF)-Supportive Framings.

Framing	Illustrative Quotation
Misuse, Abuse, and Diversion	“While [OxyContin] was safe and effective, it did have an Achilles heel, a specific vulnerability. The vulnerability that we didn’t anticipate, that could track and lead to the type of abuse and outcomes that we heard about just a few minutes ago, was that it could be easily crushed … To abusers who were seeking an opioid, that was terrific. To patients, thankfully far less often than abusers, it could also mean a bad outcome”.^37^
Crime and the Streets	“Criminal enterprises have far too easy a time diverting legitimate pharmaceuticals to illicit street drugs. This is because the most common forms of opioid-based drugs are easily manipulated”.^38^
The Need to Remove Generic Controlled-Release Oxycodone from the Market	“…the generic oxycodone recently became available in Canada, and Health Canada approved new generic formulations of it. These new formulations are addictive, and they are not tamper-resistant. As such, they will impact First Nations that are struggling with this ongoing addiction”.^39^
Medical Breakthroughs and Technological Innovation	“There is strong scientific confirmation of tamper-resistance technology and abuse-deterrent formulations. There are no maybes or guesswork about these technologies. They work. Tamper-resistance and abuse-deterrent formulation technology is here to stay. It is sound science. It is the future . . . . These technologies are a product of the scientific advances of the western pharmaceutical technology”.^38^

**Table 2.** Abuse-Deterrent Formulations (ADF)-Oppositional Framings.

Framing	Illustrative Quotation
Mistrust of Purdue	“Your company, Purdue, did the exact same thing with oxycodone/OxyContin, sending out an army of detail reps to persuade thousands of doctors with no clear evidence that it was safer than heroin or morphine, and it wasn’t really addictive. Now you’re here today doing the same thing for OxyNEO”.^37^
Concerns About Promotion of Opioids	“Purdue is now marketing [OxyNEO]. The generics do not market or promote their product to doctors”.^40^
Skepticism of Evidence	“I’ve been hearing reports that [ADFs] are fairly easy to circumvent and that you can microwave them and then inject the substance. Are there ways to circumvent these tamper-resistant drugs? If not, don’t addicts just switch to some other drug?” ^37^
Health Canada's Mandate Limitations	“Our framework is as it is. We do look at risk. We look at risks to intended patients, the ones that the drug is supposed to be used by, and we ensure that it's labelled properly”.^41^

### ADF-Supportive Framings

#### Misuse, Abuse, and Diversion

Supportive stakeholders repeatedly framed the overdose crisis as an issue of misuse, abuse, and diversion. Abuse and misuse were thus frequently equated with “extra-medical use” that included all use outside of a prescription's parameters. This narrative was exemplified in testimony from Purdue CEO Craig Landau, who situated the problems associated with OxyContin as unintentional and a consequence of an “Achilles heel”—their crushability—thereby providing an opportunity to frame Purdue as genuinely surprised by and concerned about the crisis. This framing positioned “abusers” and tampering against “patients” and legitimate use, continuing the dichotomy between the two groups.

These framings consequently created two types of opioid use, associating ADFs with Purdue, legitimacy, medical validation, patients, innovation, and proper use; and non-ADF oxycodone with street dealers, illicit use, street use, abusers, crime, and diversion. This logic consequently positioned Purdue, the maker of OxyContin ADFs, as well-intentioned, medically oriented, and legitimate.

In tandem, proponents consistently defined the policy problem as an issue of opioids being used in unintended ways. Again, abuse and misuse were repeatedly equated with tampering, reinforcing the perception that extra-medical opioid use primarily consisted of tampering. Yet unlike the committees’ more heterogenous framings, MPs conveyed these problems with notably consistent language, repeatedly using the phrase “misuse, abuse, and diversion.” These three terms, often in the exact same order, came up repeatedly in communications from supportive stakeholders, as echoed by MP Kevin Sorenson: “[ADFs] would make these drugs more difficult to crush, snort or inject, and reduce their potential for misuse, abuse, and diversion to our streets by criminals”.^38^ This framing of the crisis allowed proponents to position “abuse” as a catch-all term for extra-medical use, non-intended use, non-prescribed use, and various forms of tampering condemned by policymakers. This positioning directed attention to extra-medical opioid use as a matter of individual irresponsibility, while evading broader issues of addiction or problematic use through standard oral administration. By consistently pairing “abuse and misuse” with “diversion”—a criminal activity unrelated to actual use—proponents further reinforced the association of abuse and misuse with criminal behaviour. This binary framing may have also served as a form of symbolic reassurance to the public, signaling that the government was protecting “legitimate patients” while advancing punitive measures against criminal activities.

#### Crime and the Streets

In addition to and often in tandem with attributing the crisis to abuse, misuse, and diversion, proponents consistently framed their support by referring to concerns regarding crime and the streets. Proponent MPs framed the legal and illegal opioid markets as, respectively, “legitimate” and “illegal” or “street drugs,” as seen in Conservative MP Rachel Thomas's testimony: “Criminal enterprises have far too easy a time diverting legitimate pharmaceuticals to illicit street drugs. This is because the most common forms of opioid-based drugs are easily manipulated”.^38^ MP Thomas's reference to streets was echoed by MP Sorenson, who noted that ADFs would “. . . reduce [opioids’] potential for misuse, abuse, and diversion to our streets by criminals”.^38^ These framings thus again fortified one another, connecting *tampering* with *abuse*, and *abuse* with *criminality* and *streets.*

Supporters furthered this “criminality” framing by referring to stories of people who sold their prescriptions. MP Sorenson, for instance, brought up diversion, although he—possibly inadvertently—acknowledged that reselling of prescriptions was occurring with tamper-resistant products as well, by noting that OxyNEO pills were also being re-sold: “In New Brunswick . . . It is alleged the 35-year-old doctor wrote prescriptions for 50,000 OxyContin and OxyNEO pills, picked them up herself, and did not give them to the patients”.^38^ Proponents implied that making opioids harder to abuse would create a smaller market for diversion. Others argued that individuals were becoming addicted to “street drugs” by first using illegally tampered “legitimate” opioids. In other instances, those who were tampering with the products were referred to as criminals. Throughout, proponents attempted to connect supporting the bill with fighting criminality more generally. Proponents thus played on a “tough on crime” sentiment, yet only a specific type of crime—diversion. Other criminal activities—such as from industry itself—were left untouched by this framing.

In positioning non-ADF oxycodone as synonymous with illegal drugs and crime, while positioning ADF oxycodone as meeting a medical need, supporters of mandating ADF formulations fostered a dichotomous framing between legitimate, medical use and deviant and illegal use. Liberal MP Fry, Liberal Senator Eggleton, and Conservative Senator Ogilvie repeatedly framed non-ADF opioids in terms related to “street use” and heroin. This was summarized by Senator Ogilvie: “Testimony has indicated there is a new form that makes it much more difficult for that drug to be used as a narcotic in the normal sense of that term . . . why haven’t we banned a legalized form of heroin?”^42^ By referencing heroin, the Senator drew attention to a drug with no regulator-approved prescription use. Ogilvie equates using narcotics “in a normal sense” to taking them recreationally, rather than by prescription. Purdue CEO Landau's discussion of “abusers” also often came in tandem in discussions of diversion and crime. He spoke of ADFs reducing the potential for criminal activities related to opioids, stating: “If a product is less attractive to abusers, it could mean less doctor-shopping, less diversion, and less theft or criminal activities related to obtaining it . . . .”^39^ The specific allusions to streets and heroin thus continued the dichotomy between “legitimate” and “illicit” use, contrasting “medical” and “street” use. The Senators’ particular emphasis on oxycodone as “effectively heroin” highlights this dichotomy; simply being “like heroin” was itself considered problematic.

Much of this framing was also articulated in relation to the routes by which oxycodone itself was taken—Senator Eggleton, for instance, noted his concern that oxycodone was “crushable and useable on the street.” This framing thus continued the “abuse and misuse” framing through the dichotomy between “legitimate” and “criminal” or “street” users. Overall, these framings safely associated ADFs with legitimacy and a medical model, while oxycodone was consistently placed next to criminality, tampering, and abuse.

#### The Need to Remove Generic Controlled-Release Oxycodone from the Market

Supporters focused on removing generic oxycodone rather than emphasizing ADFs’ value. For instance, Phil Emberley, Director of Pharmacy Innovation for the Canadian Pharmacists Association (CphA), specifically noted that stocking crushable forms of generic oxycodone left pharmacies more vulnerable to robberies. Peter Dinsdale, Chief Executive Officer of the Assembly of First Nations, framed mandating ADFs as a way to remove drugs that disproportionately, negatively influence First Nations communities: “. . . the generic oxycodone recently became available in Canada, and Health Canada approved new generic formulations of it. These new formulations are addictive, and they are not tamper-resistant. As such, they will impact first nations that are struggling with this ongoing addiction”.^39^ Notably, there was no difference in generic and brand-name oxycodone's risk for addiction. Yet these characterizations combined advocacy for tamper resistance with broader concerns about the widespread availability stemming from OxyContin coming off patent. The main problem was therefore defined as continual access to generic controlled-release oxycodone that could be altered or modified, which mandating ADFs could eliminate. This shared definition resulted in a heterogenous policy coalition of vocal supporters with significantly different rationales, but all concurring that ADFs would discourage misuse of controlled-release oxycodone. This framing served to define the problem as accessibility of opioids (rather than tampering), with ADFs serving as a means to reduce this accessibility.

#### Medical Breakthroughs and Technological Innovation

Compared to the relatively measured language in the House and Senate committees, supportive MPs’ language was far more effusive. This contrast may reflect MPs’ political incentives to appear responsive and decisive in the face of rising public concern, leading them to embrace the language of technological optimism and frame ADFs as urgent and innovative solutions. In framing ADFs as both innovative breakthroughs and the outcome of significant investment, proponents promoted a broader discourse that industry has sought to perpetuate for decades: one of significant innovation on account of large investments into novel products. When introducing Bill C-307, for instance, MP Sorenson repeatedly spoke of his amazement at the technology: “Tamper-resistant technologies are evolving fast, and these advancements in medical science are absolutely exciting . . . .”^38^ Supportive MPs repeatedly referred to the innovation and technology when discussing ADFs, which was echoed in MP Mackenzie's statement of support: “There is strong scientific confirmation of tamper-resistance technology and abuse-deterrent formulations. There are no maybes or guesswork about these technologies. They work. Tamper-resistance and abuse-deterrent formulation technology is here to stay. It is sound science. It is the future . . . . These technologies are a product of the scientific advances of the western pharmaceutical technology”.^38^

While emphasizing the crisis as resulting from individuals using OxyContin in ways that were not originally intended by the industry, ADF proponents simultaneously posed the pharmaceutical industry as an innovative actor that could help to solve a crisis for which the industry bore no responsibility. By shifting the framing of the problem and its causes from industry to individuals, stakeholders positioned the industry as a central player in addressing opioid-related issues. With the problem defined as individuals bypassing the opioids’ safety mechanisms, proponents promoted the solution of innovative approaches that would make abuse more difficult.

### Oppositional Framings

#### Mistrust of Purdue

Two stakeholders framed their opposition to ADFs out of a distrust of Purdue and the sincerity of their intentions. University of Toronto Family and Community Medicine professor Nav Persaud and Conservative MP Terence Young voiced their skepticism of ADFs in principle due to Purdue's complicity in the crisis more generally (MP Young's daughter died from a prescription drug and MP Young has been a notable advocate for prescription drug reform^43^;). Persaud was brought into the Senate committee hearings to speak about improving pharmaceutical regulatory systems. In the course of his testimony, Persaud was asked about Landau's claims and Persaud expressed deep skepticism considering the background of Purdue's past actions: “Regarding the newer formulation, it's remarkable, given the history—that is, the history of Purdue illegally making the claim in the United States that its product, OxyContin, which was the newer formulation at the time, had a lower abuse potential—that still today Purdue seems to be singing the same tune; i.e., saying that this even newer formulation that they have today has a lower abuse potential. I think that it's likely to result in exactly the same problem”.^1^ Persaud's logic mirrored CPC MP Terence Young's, who repeatedly interrogated Purdue CEO Landau about his company's motivations. Explicitly noting Purdue's profit incentives surrounding ADFs and track record of unethical marketing practices, MP Young noted, “Your company, Purdue, did the exact same thing with oxycodone/OxyContin, sending out an army of detail reps to persuade thousands of doctors with no clear evidence that it was safer than heroin or morphine, and it wasn’t really addictive. Now you’re here today doing the same thing for OxyNEO”.^37^

Persaud and Young's opposition was framed in terms of Purdue's track-record of misleading the public and policymakers, rather than any particular opinion on ADFs themselves. The overall message that emerged was that the same industry that caused the problem could not be the one to fix it. Yet, it appears that this sentiment was only felt among a small number of stakeholders, with most others willing to permit Purdue to benefit if it meant abating the crisis. This willingness to align themselves with Purdue may reflect a form of pragmatic alignment, in which stakeholders accepted Purdue's involvement not because they trusted the company, but because ADFs offered a politically palatable intervention that avoided more contentious structural reforms.

#### Concerns Regarding Promotion of Opioids

Representatives from the Canadian Generic Pharmaceutical Association (the trade association for the generic pharmaceutical industry) framed their opposition to mandating ADFs through the fact that generic drug manufacturers do not promote their products, unlike Purdue, and that generic prices are cheaper. With the role of aggressive promotion of opioids emerging as a root cause of the crisis, their logic was that mandating ADFs would allow Purdue new intellectual property with OxyNEO and consequently re-permit promotion: “Purdue is now not marketing [OxyNEO]. The generics do not market or promote their product to doctors. If a doctor has a patient stabilized on OxyContin and wants to continue to use that, then the generic is there and available at the much lower price, typically, at which generics are sold. It is dispensed and supplied throughout the supply chain in a very safe, effective way”.^40^ The Canadian Generic Pharmaceutical Association's opposition to ADFs was thus framed in terms of the need to reduce the marketing and promotion of opioids more generally. This argument is applicable to most generic medicines in comparison to brand-name medicines because brand-name medicines often undergo extensive marketing campaigns and branding efforts, whereas generic medicines typically do not have the same level of promotional activities.^44^ This framing, however, had a particular strength in light of Purdue's more general misdeeds, thus building on Persaud and Young's framings and effectively leveraging the poor reputation of Purdue once again.

#### Skepticism of Evidence

A primary form of opposition to ADF mandates was framed in terms of their ineffectiveness in reducing harm. The arguments on effectiveness had two main foci: users’ ability to circumvent the technology and users switching to other products, as CPC MP Eve Adams noted: “I’ve been hearing reports that [ADFs] are fairly easy to circumvent and that you can microwave them and then inject the substance. Are there ways to circumvent these tamper-resistant drugs? If not, don’t addicts just switch to some other drug?”^37^ These framings re-entrenched the notion that tampering was a primary concern. Instead of disputing this notion, they disputed the effectiveness of ADFs to *address* this issue. Thus, this counter-framing allowed for the abuse and misuse framing to remain. This highlights the strength of the abuse and misuse framing, as it forced opponents to provide evidence of ADFs’ ineffectiveness, rather than disputing the actual notion of abuse and misuse as itself a primary cause of the crisis.

#### Health Canada's Mandate Limitations

Some of the strongest opposition to mandating ADFs with Health Canada came from Health Canada itself. During the Senate committee, Supriya Sharma, Acting Associate Assistant Deputy Minister, Health Products and Food Branch (Health Canada), was questioned as to why generic versions of OxyContin were still permitted rather than solely permitting OxyNEO. In two separate statements, Sharma provided a thorough explanation that framed Health Canada's approval of oxycodone as constrained by their own mandate to simply establish drug equivalency and bio-equivalency. Sharma also positioned ADFs through a “risks and benefits” framing, placing the risks of evasion as outweighing the benefits of any improved health outcomes. Sharma specifically noted an absence of known diversion of generic oxycodone. Sharma's statement foreshadowed a more general framing of opposition to mandating ADFs—citing evidence for evasion of the tamper-resistant features and the health risks faced by those who still attempt to tamper with the product, as well as the lack of evidence for high diversion of generic forms of OxyContin. This perspective was also reflected by John Patrick Stewart, senior executive director, Therapeutic Products Directorate: “Our framework is as it is. We do look at risk. We look at risks to intended patients, the ones that the drug is supposed to be used by, and we ensure that it's labelled properly”.^41^ Stewart presented Health Canada's decision-making process as constrained by its own mandate. Decisions that fell outside of the mandate—that is, abuse by those for whom opioids were not intended—were therefore not their area of focus. Stewart later spoke similarly of being constrained when discussing the inflexibility of the Canadian system in comparison to the United States's system, stating the United States “have some flexibility in the way they can evaluate the evidence, and evidence that might be related to abuse deterrents outside of the intended population. Under our framework in Canada, we have to focus on the intended use”.^41^

Overall, Health Canada members largely framed their decisions as constrained by their own mandate to only look at *technical* evidence, rather than the broader societal context. Their statements primarily highlighted technical and legal constraints, effectively arguing for what Health Canada could do, rather than what they should do. This framing allowed Health Canada to defer responsibility to the government, effectively absolving themselves of responsibility to act.

## Discussion

### Summary of Findings

Debates over ADF mandates reflected competing interests and problem definitions in addressing the opioid crisis. Proponents emphasized removing generic oxycodone, framing ADFs as innovative solutions to curb misuse, diversion, and tampering, while associating non-ADF opioids with street use and illegitimacy. Conservative MPs and supporters portrayed ADFs as medical breakthroughs essential for addressing prescription opioid harms and preserving legitimate medical use. Opponents, however, expressed skepticism about ADFs’ effectiveness, mistrust of Purdue due to its role in the crisis, and concerns about Health Canada's mandate and potential unintended consequences. They argued for maintaining non-ADF opioids to provide a safer supply for those already addicted, signaling a recognition of prescription opioids’ dual role in treatment and harm reduction.

ADF debates influenced how the overdose crisis is understood, the role of industry in addressing it, and the broader societal narratives about opioid misuse and addiction. By framing the crisis in terms of individual misuse, tampering, and criminality, ADF proponents reinforced a narrow understanding of the problem that diverted attention from systemic issues such as overprescription, socioeconomic determinants, and the culpability of pharmaceutical companies. These narratives legitimized ADFs as a viable solution, despite mixed evidence of their effectiveness and their potential to exacerbate harms by driving users to illicit markets. Supportive language in these debates served to entrench a dichotomy between “legitimate” opioid use and “illegitimate” misuse, reinforcing stigmatizing narratives that framed individuals as the primary culprits of the crisis while absolving systemic contributors, such as overprescription and industry practices. By tracing how these framings defined misuse in narrow terms, minimized addiction, and overlooked structural contributors to harm, this study illuminates how policies, even when not implemented, contribute to the normalization of particular understandings of public health crises.

### Abuse, Misuse, and Addiction

Our findings build upon critiques framings of the opioid crisis in terms of individual misuse and criminality while minimizing systemic contributors to harm.^45,46^ Simplistic framings around “abuse, misuse, and addiction” risk diverting attention from systemic issues like overprescription, socioeconomic determinants, and the culpability of pharmaceutical companies. By reinforcing a dichotomy between “legitimate” opioid use and “illegitimate” misuse, these framings perpetuate stigmatizing narratives and absolve systemic contributors from responsibility. While the distinctions between abuse, misuse, and addiction may appear subtle, they come against the backdrop of historical downplaying of addiction. Purdue had first marketed OxyContin as having “lower potential for addiction”,^47^ which was categorically false. Conflating these terms obscured OxyNEO's inability to address addiction itself, leading to potential misunderstandings of OxyNEO's ability to address a root cause of the crisis. A key finding here is thus the way in which opioid use, misuse, abuse, and extra-medical use quickly became conflated. In blurring the lines between these definitions, actors’ framings frequently consolidated people with opioid use disorders, those using opioids outside of the products’ intended medical practice, and those tampering with opioids to gain a more immediate “high.” By distinguishing opioids between “legitimate” and “illegal,” supporters framed the crisis as misuse, absolving Purdue of responsibility.

These framings were further enabled by a permissive regulatory environment. Health Canada's regulatory approach, for instance, enabled Purdue to include claims of reduced abuse potential in OxyContin's Product Monograph, despite insufficient supporting evidence.^48^ This reflected a broader regulatory tendency to focus narrowly on labeling and technical compliance rather than proactively interrogating the public health implications of marketing claims.^49–51^ By allowing unverified messaging to appear in official documentation, Health Canada helped legitimize a narrative that downplayed addiction risks, contributing to the normalization of aggressive opioid promotion. This example highlights how regulatory silence or inaction can enable problematic industry framings, even in the absence of overt endorsement.

### Clinical Models and Policy Frames

While policymakers and industry actors frequently framed opioid use within a binary of “legitimate patient” versus “illicit abuser,” this dichotomy risks oversimplifying the complex realities of opioid consumption. For example, the Diagnostic and Statistical Manual of Mental Disorders, Fifth Edition (DSM-5), defines *opioid use disorder* (OUD) as a problematic pattern of opioid use leading to clinically significant impairment or distress, as manifested by at least two of eleven criteria within a 12-month period.^52^ These criteria encompass behaviors such as unsuccessful efforts to cut down or control opioid use, craving, and continued use despite social or interpersonal problems caused by the effects of opioids. This is one example of how opioid use can be conceptualized on a spectrum of severity and independently of the legality of the substance. Consequently, this definition acknowledges that tolerance and withdrawal, which are often associated with physical dependence, are not sufficient for an OUD diagnosis when opioids are taken under appropriate medical supervision. But, it also highlights that patients adhering to prescribed regimens can still develop OUD, challenging the notion that misuse or abuse is solely a matter of illicit or non-medical use. Of interest, clinical frameworks were almost entirely absent from the policy debates, where discussions rarely referenced diagnostic criteria or the spectrum of opioid-related disorders.

### Framing “Innovation”

Framing ADFs as innovations aligned with the pharmaceutical industry's strategy to maintain market exclusivity,^53^ a critical concern given that the perception of innovation is closely tied to a product's ability to secure exclusivity.^54^ As such, pharmaceutical manufacturers have increasingly relied on minor changes to drug formulations or alterations in drug manufacturing that are neither significantly innovative nor clinically meaningful, consequently providing manufacturers with patents that block competition and permit market exclusivity.^55^

Proponents’ “innovation” framing was thus overall consistent with a broader turn in the pharmaceutical industry to framing as “innovation” companies, rather than “pharmaceutical” companies; in 2016, while ADFs were being framed as technological innovations, the Canadian organization representing the majority of Canadian pharmaceutical companies rebranded itself as “Innovative Medicines Canada” in 2016, changing its name from Canada's Research-Based Pharmaceutical Companies (Rx&D).^56^ ADFs thus also served as an opportunity—on a public stage—for the industry to highlight itself as “innovative” during a crisis of innovation and a rebranding campaign.

### The Opioid Industry and Unlikely Overlapping Framings

While this study represents a contribution to the wider literature on framing, it also serves as an important addition in its emphasis on how a diverse framing coalition of actors, many of whom do not necessarily appear favourable to the industry, come to support an industry-favoured solution. Stakeholders defending ADF mandates repeatedly framed the crisis in relation to misuse and abuse. This framing reproduced Purdue's own problem definitions surrounding the crisis, particularly regarding fostering a dichotomy between “legitimate” and “illegitimate” opioid users. While many ADF supporters took pains to avoid supporting Purdue—such as when NDP MP Don Davies, despite supporting the mandate, expressed skepticism of Purdue's genuine interest in addressing opioid-related harms—they uncritically replicated industry framings and supported policies that favoured Purdue.

While ADF proponents often aligned with industry-friendly framings, it would be overly simplistic to label all advocates as industry-friendly shills. Some policymakers may have adopted these framings not out of ideological alignment with industry but because they resonated with broader political commitments—such as balancing public safety with access to pain treatment or maintaining credibility with both medical and law enforcement constituencies. Analyses of industry influence must therefore consider the broader sociopolitical determinants shaping policy support. By contextualizing this framing within the sociopolitical landscape, this study aims to challenge the common perception of “industry interference” as a straightforward phenomenon.

These findings thus highlight that support for ADFs largely aligned with policy actors’ own interests rather than perspectives on the opioid industry itself. These debates should therefore not be viewed as mere disagreement between industry and public health but rather between two sets of heterogenous coalitions with vastly different reasons for supporting or opposing a mandate. While Purdue stood to *benefit* from the mandate, most supporters’ communications implied that their own interests in a mandate were largely unrelated to the industry's. Instead, these findings underscore how policy actors may oppose or support industry-favoured solutions independently of their views on the industry.

Given the robust evidence that overdose crisis framings shape public attitudes toward stigma, treatment, and the attribution of blame,^32,57,58^ framings that position manufacturers as solution-providers and ADFs as technological fixes are likely to have significant consequences in deflecting scrutiny and absolving the industry of responsibility.

### Strengths and Limitations

There are numerous strengths to this study. First, the diversity of stakeholders involved in the ADFs policy arena permits cross-stakeholder analysis that reveals the heterogenous framing coalitions that formed around particular policy solutions. Second, analyzing framings across three policy arenas provides an opportunity to observe how framings evolved temporally. Third, this study provided a clearly needed analysis of how the opioid industry benefits from particular framings. Despite numerous studies of industry's own framings,^35,59,60^ there has been minimal effort to analyze how the pharmaceutical industry *benefits* from other policy actors’ discourses.

There are also a number of limitations to this study. First, we had access only to public communications and conversations. These resources solely consisted of public discussions in which speakers were aware that they were speaking on record. This awareness inherently limited the breadth and depth of the available data, leading to a skewed representation of actors’ true sentiments. Conversations that occurred in less accessible spaces—such as meetings between Purdue and MPs—were simply unavailable. As a result, it cannot be fully known how or why members eventually came to support or oppose the mandate.

Second, while this study analyzed the framings articulated in formal public discourse, it did not examine how individual stakeholders’ positions may have been influenced by financial relationships with the pharmaceutical industry or broader political ideologies. Investigating such influences would require access to financial disclosures, private communications, or internal party strategies—data sources beyond the scope of this framing analysis. Future research might fruitfully explore how structural political alignments or industry relationships shaped the uptake of particular opioid-related narratives.

Finally, interpretive policy research inherently involves both subjective presentations by policy stakeholders and interpretation by the researcher—commonly termed the *double hermeneutic*.^61^ It is therefore possible that others would assign different meanings to the same texts. Notwithstanding, particular attention was paid to multiple readings and aiming to ground this analysis in intended meanings. Given that this analysis is also part of a broader study on the topic, this wider knowledge of the relevant content strengthens the validity of the analysis.^10^

## Conclusion

Policy stakeholders employed a wide range of framings during debates surrounding ADF opioid mandates in Canada. Our findings suggest that ADF mandates served as proxy for broader disagreements regarding the harms of prescription opioids, the overdose crisis, the culpability of the opioid industry, and who should be considered a “legitimate” opioid patient. This analysis thus provides important insights into how policy stakeholders were able to use a policy debate focused on tamper-resistant technologies to frame the causes of and solutions to the crisis more generally.
